# Sources, Extraction and Biomedical Properties of Polysaccharides

**DOI:** 10.3390/foods8080304

**Published:** 2019-08-01

**Authors:** Samee Ullah, Anees Ahmed Khalil, Faryal Shaukat, Yuanda Song

**Affiliations:** 1 Colin Ratledge Center for Microbial Lipids, Center for Functional Foods and Health, School of Agriculture Engineering and Food Science, Shandong University of Technology, Zibo 255049, China; 2 University Institute of Diet and Nutritional Sciences, Faculty of Allied Health Sciences, The University of Lahore, Lahore 54000, Pakistan

**Keywords:** bioactive polysaccharides, extraction, biomedical applications

## Abstract

In the recent era, bioactive compounds from plants have received great attention because of their vital health-related activities, such as antimicrobial activity, antioxidant activity, anticoagulant activity, anti-diabetic activity, UV protection, antiviral activity, hypoglycemia, etc. Previous studies have already shown that polysaccharides found in plants are not likely to be toxic. Based on these inspirational comments, most research focused on the isolation, identification, and bioactivities of polysaccharides. A large number of biologically active polysaccharides have been isolated with varying structural and biological activities. In this review, a comprehensive summary is provided of the recent developments in the physical and chemical properties as well as biological activities of polysaccharides from a number of important natural sources, such as wheat bran, orange peel, barely, fungi, algae, lichen, etc. This review also focused on biomedical applications of polysaccharides. The contents presented in this review will be useful as a reference for future research as well as for the extraction and application of these bioactive polysaccharides as a therapeutic agent.

## 1. Introduction

Polysaccharides are considered as vital bio-macromolecules for all living organisms, which are structurally comprised of homo or hetero monosaccharides and uronic acids connected with glycosidic linkages [1,2,3]. They are predominantly found in various parts of plants, animals, fungi, bacteria, and seaweed, and play a critical role in numerous physiological functions of life [4]. Polysaccharides along with lipids, proteins, and polynucleotides are classified as the most pivotal four macromolecules in life sciences. Bioactive polysaccharides are known as polysaccharides produced from living organisms, and/or are functionalized from sugar-based materials possessing biological effects on organisms. Moreover, during the last decades, bioactive polysaccharides have been investigated as therapeutic agents against many chronic diseases due to their biodegradability, non-toxic nature, and biocompatibility [5]. Studies have shown that polysaccharides possess a wide range of pharmacological perspectives such as antioxidative, antitumor, antimicrobial, anti-obesity, hypolipidemic, antidiabetic, and hepato-protective properties [6,7,8]. They have been investigated extensively for the development of novel products in the field of cosmetics, food, medicine, petrochemicals, and paper [3,9,10]. Particularly, in the medicinal industry, polysaccharides are mostly used as pharmaceuticals and medical biomaterials (hypoglycemic, anti-osteoarthritic, and anticancer products) to curtail the effect of respective metabolic syndromes [9,11].

The potentiality of bioactive polysaccharides is strongly influenced depending upon their configuration and chemical structure. Nevertheless, the macromolecular configurations of plant cellular polysaccharides, particularly hetero polysaccharides (hemicelluloses), are very complex owing to the occurrence of various monosaccharides acting as isobaric stereoisomers [12,13]. Additionally, polysaccharides present in plants, microorganisms (bacteria, fungi, and yeasts), animals, and algae are chemically and/or physically bound with various other biomolecules like lignin, proteins, lipids, polynucleotides, and a few minerals [14]. Hence, to understand the importance of bioactive polysaccharides in the domain of life sciences resulted in the multi-disciplinary collaboration of scientists from the fields of microbiology, phytology, glycol-biology, nutrition, food sciences, and glycol-medicine [5]. Polysaccharides both in the simple and complex glycol-conjugated form are renowned for various bioactive functions, for instance, antivirus, antioxidant, antimicrobial, anticancer, antidiabetic, reno-protective, immunomodulatory, and anti-stress perspectives.

## 2. Sources of Bioactive Polysaccharides

Bioactive polysaccharides are categorized broadly depending upon their sources, structure, applications, solubility, and chemical composition. On the basis of chemical composition, they are characterized as homopolysaccharides (homoglycans) and heteropolysaccharides (heteroglycans). Homoglycans comprise of a single type of monosaccharide, such as glycogen and cellulose which consists of glucose molecules, whereas heteroglycans are made up of a different type of monosaccharides, for example, heparin and chondroitin sulfate (CS) [15]. 

According to the glycosides linked on to the glycan, polysaccharides can also be classified as proteoglycans and glycoproteins, glycolipids, and glycoconjugates [16,17]. According to grouping based on origins, bioactive polysaccharides are usually classified as those derived from animals (chondroitin sulfate, heparin, and hyaluronan), plants (pectin, inulin, Ginseng polysaccharides, xylans, and arabinans), bacteria (exopolysaccharides, capsular polysaccharides, and peptidoglycans), lichen, fungi, and algae. In the section below, bioactive properties associated with polysaccharides are reviewed depending upon their natural sources in order to understand their functionality. 

### 2.1. Bioactive Polysaccharides in Dietary Fibers

The FAO (Food and Agriculture Organization) defined dietary fibers as a variety of indigestible plant polysaccharides including pectins, cellulose, gums, hemicelluloses, oligosaccharides, and various lignified compounds. Dietary fibers are the type of polysaccharides that are biologically active in their innate form and/or after enzymatic and chemical treatments. For instance, celluloses and hemicelluloses directly stimulate the bowel movement, whereas inulin is fermented by microflora with the intent to prevent various gastrointestinal disorders [18]. Dietary fibers play a vital role in prevention from various diseases especially hyperlipidemia, cardiovascular complications, and obesity. Gums, inulin, and pectin are capable of slowing down the passage of food in the digestive tract, reducing serum cholesterol concentrations, hindering the absorption of sugar from food to bloodstream and, therefore, avoiding sudden hyperglycemic conditions post food ingestion. 

Among dietary fibers, lignin, cellulose, and hemicellulose are classified as insoluble fibers which help in the stimulation of the bowel movement, speeds up the removal of waste via the digestive tract, and helps in prevention of hemorrhoids, constipation, and diverticulosis [19,20]. Results of various epidemiological investigations and clinical trials have suggested that consumption of moderate or higher levels of dietary fiber efficiently reduces the risk of numerous metabolic syndromes like diabetes [21], strokes [22], hypertension [23,24], hyperlipidemia/hypercholesterolemia, [25,26], obesity, and cancer [27]. Usually, ingestion of dietary fiber has health modulating benefits, like increased stool bulk, reduced intestinal transit time, a decline in levels of total cholesterol (TC) and postprandial blood glucose, and/or insulin contents [27,28,29]. Keeping in view the significance of dietary fibers with special reference to human health and their associated mechanism of actions, these are considered as an abundant source of bioactive polysaccharides. Table 1 summarizes the types, names, sources, composition, and physiological effects of dietary fiber polysaccharides from various studies and trials. 

### 2.2. Bioactive Polysaccharides in Herbs

Herbs have attained a significant position in traditional medicines (Indian Ayurveda, ancient Chinese medicine, phytomedicine in western countries, and Japanese Kamp medicine) of numerous countries owing to their associated curing benefits against numerous diseases. Outcomes of recent pharmacological studies have illuminated that the chief component of herbal medicines usually comprises of tannins, polysaccharides, high molar mass proteins, and low molar mass constituents like terpenoids (quassinoids, sesquiterpenes, and *Rabdosia* diterpenes), saponins, alkaloids (protoberberine alkaloid, phenanthridine, etc.), and flavonoids (*Scutellaria* flavones) [30]. Among the above-mentioned compounds in herbal medicines, polysaccharides are believed to be main bioactive compounds responsible for various pharmacological potentials like antitumor, antioxidant, hepato-protective, antiviral, radio-protective, immuno-stimulatory, and anti-fatigue activities [31,32,33,34,35]. Innate polysaccharides present in numerous herbs are known for stimulation of the human immune system, inhibition of viral replication, scavenging free radicals, and inhibition of lipid peroxidation [33,36,37]. Recent advancements regarding the application of polysaccharides present in herbal medicines for prevention and cure of diseases are documented in Table 1.

### 2.3. Bioactive Polysaccharides in Algae and Lichens

Polysaccharides present in lichens and algae have been of great interest to the food scientists owing to their associated exceptional physical properties (stabilizing, gelling, and thickening capability) and biological potentials (immunomodulating, antiviral, anticoagulant, antitumor, antioxidative, anti-inflammatory, and anti-thrombotic activity) [48,49]. Basically, sulfated polysaccharides are the main group of the most attractive and interesting constituents in marine algae i.e., laminarans and fucoidans in the Phaeophyceae (brown algae), carrageenans in the Rhodophyceae (red algae), and ulvans in the Chlorophyceae (green algae) [40]. One of the most vital properties linked with sulfated polysaccharides is their anticoagulant characteristics. For instance, carrageenan (sulfated galactans) isolated from red algae and sulfated fucoidans extracted from brown algae are known to possess excellent anti-coagulant properties. Sulfated polysaccharides identified in algae have been stated to own equal or even stronger activities than those linked with heparin [32]. Various other biological properties of sulfated polysaccharides present in algae have been intensively investigated.

Experimentally, fucoidan has been reported to possess maximum antioxidative properties followed by alginate and laminaran, therefore protecting human health from the damage of ROS (reactive oxygen species). Likewise, the anticancer and anti-tumor characteristics of algae-based sulfated polysaccharides are found to be due to their antioxidative and free radical scavenging properties [50]. Moreover, inhibition of HIV and herpes viruses in cells owing to anti-viral potential of sulfated rhamnogalactans, carrageenans, and fucoidans have also been scientifically proven [40]. Immuno-modulating characteristics associated with algae-based sulfated polysaccharides were demonstrated by increasing the secreting and phagocytic activities of macrophages [51]. 

Polysaccharides derived from lichens (composite organism formed due to a symbiotic partnership among alga and fungus) are principally linear or rarely substituted α-glucans and/or β-glucans. This type of glucans isolated from lichens is reported to have a wide range of bioactive functions such as immunomodulatory perspectives, anti-tumor potential, and anti-viral characteristics [49,52,53]. The immuno-modulating activity of polysaccharides (β-glucans) derived from lichenin and lichens are linked to stimulation of a variety of immune responses like production of NO (nitric oxide) and ROS (reactive oxygen species) and release of cytokines and arachidonic acid metabolites [51]. Lichenin, also known as lichenan, consists of a galactoglucomannan (water-soluble hemicellulose) structure that is responsible for anti-thrombotic and anti-coagulant properties [54]. Bioactive properties related to sulfated polysaccharides from lichens and algae are highly dependent on their structural properties, for instance, sulfate concentration and distribution of sulfate groups on the main chain, stereochemistry, and molar masses. Hence, there is a need for the modification of innate sulfate polysaccharides to attain biologically active polysaccharides having desired molecular size and functionality for bioactive further application [40,55]. An overview of some recent advancements in this context are depicted in Table 2.

### 2.4. Bioactive Polysaccharides in Fungi

A wide range of bioactive saccharides is present in fungi ranging from monosaccharides to polysaccharides. They can be summarized as the intracellular or extracellular polysaccharides depending upon their presence in the fungi [56]. The bioactive polysaccharides derived from basidiomycetes are reported to have antitumor and anticancer activities and can prevent breast cancer in post-menopausal women. The compound which can inhibit tumors was first isolated from the basidiomycete *Boletus edulis* in 1957. In traditional medicine, several basidiomycete mushrooms are used as an immunosuppressant to treat cancer and even for treating AIDS [57]. Polysaccharopeptide (PSP) isolated from *Ganoderma lucidum* induces the inhibition of oncogenes and kill the tumor cells directly. Similarly, the bioactive polysaccharides schizophyllan, lentinan, and polysaccharide-K isolated from *Grifola frondosa* and *Trametes versicolor*, respectively, improve the immune function of the host and showed a synergistic effect with chemotherapy [58]. Just like mushrooms, another source of bioactive polysaccharides is *Cordyceps militaris*, and they possess a wide range of structurally different bioactive polysaccharides. Water-soluble polysaccharides from *C. militaris* contain a (1 → 4) galactose linkage and (1 → 3,6)-mannose linkage and branching usually arising from (1 → 4)-linked glucose. These polysaccharides were reported for their numerous biological activities including anti-tumor, immunomodulatory, antioxidant, and anti-inflammatory activities [56,59].

### 2.5. Bioactive Polysaccharides from Bacteria

Bacteria are a rich source of biologically active polysaccharides. On the base of structural properties, bacterial polysaccharides can be defined into five major classes: Exopolysaccharides (EPSs), capsular polysaccharides (CPSs), lipopolysaccharides (LPSs), peptidoglycans, and teichoic acids [60]. 

A lot of research has been done on hyperbranched bacterial polysaccharides (HBPSs). For example, highly branched dextran with a backbone of 75% (α-(1→6)-D-Glcp) with 19% of α-(1→3) and a few α-(1→2) branching [61], was separated from *Leuconostoc citreum* B-2 and studied extensively, and showed a water-holding capacity of up to 450% and an 80% water solubility index, consequently having potential applications in cosmetic, food, and pharmaceutical industries [62,63]. From an industrial point of view, EPSs producing Gram-negative prokaryotes (cyanobacteria) are important because of their ability to produce novel molecules. These EPSs usually linger as a sheath/capsule with the cells and when they are liberated from the cells they are termed as released polysaccharides. Some bacteria from the genus cyanobacterium, namely *Anabaena, Aphanocapsa, Cyanothece, Phormidium, Synechocystis,* and *Nostoc* are useful to produce sulfated EPSs, which also contain uronic acids [64]. Upon fermentation in the laboratory, polysaccharide yield from 0.5–4 g/L can be obtained from three known genera of marine bacteria, namely *Alteromonas* sp., *Pseudoalteromonas* sp. and *Vibrio* sp. [65]. Exopolysaccharide HE 800 is secreted by *Vibrio diabolicus* and it contains an equal amount of hexosamine and glucuronic acid, which can be depolymerized, N-deacetylated, and after chemical sulfurylation produce new derivatives of HE800. These derivatives are referred to as DRS HE800 and are structurally very close to heparan-sulfate [66]. Moreover, acidic and highly branched heteropolysaccharide (EPS GY785) is produced by the bacterium *Alteromonas infernus*. Non-saccharide units of EPS GY785 are composed of uronic acid and sugar (glucose and galactose) [67]. 

EPSs derived in the laboratory showed widespread biological activities like being an antioxidant, having cholesterol-lowering and inflammation-regulating properties, as well as anti-tumor, anti-coagulant, and antivirus activities [62,68]. Moreover, bacterial polysaccharides are used in Protein Glycan Coupling Technology (PGCT) to produce glycoconjugate vaccines against Gram-positive and Gram-negative bacteria [69].

### 2.6. Bioactive Polysaccharides in Wood

Bioactive polysaccharides present in wood primarily consists of celluloses and some primary groups of hemicelluloses (glucans, arabinans, xylans, glucomannans, and galactans) [70,71]. Pectins and galacto-glucomannans derived from wood are stated to have radical scavenging properties and immune-stimulating activities [72,73]. Xylo-oligosaccharides (xylans) derived from dietary fibers, hard and/or softwood have been reported to be used efficiently as prebiotics by nutraceutical and medicinal industries [74]. Similarly, pentosane polysulfate—a xylan derivative being extracted from beech-wood—is also known as a biomedical agent for the treatment of interstitial cystitis, a bladder pain syndrome [75]. While most polysaccharides do not reveal biological properties unless they are subjected to some modification; derivatives of cellulose-like hydroxypropyl-cellulose (HPC), hydroxypropyl-methylcellulose (HPMC), hydroxyethyl-cellulose (HEC), and methyl-cellulose have evidently proven their functional roles in numerous fields (medicinal, cosmetics, food, and pharmaceutical) [76]. 

### 2.7. Bioactive Polysaccharides from other Sources

Sulfated glycosaminoglycans (GAGs) like keratin sulfate (KS), heparan sulfate (HS), dermatan sulfate (DS), and chondroitin sulfate (CS) belongs to a class known as animal-derived bioactive polysaccharides. Heparan sulfate comprises of repeated units of N-acetylated and sulfated disaccharides (glucosamine and glucuronic/iduronic acid) units [77,78,79]. Evidently, heparin is known to be the most effectual clinical anti-coagulant used in medical sciences. The anti-coagulation potential of heparin is mainly due to its particular structural arrangement, in which specifically the binding site of anti-thrombin III (ATIII, a non-vitamin K-dependent protease) is of central importance in the prevention of fibrin clot formation which is generated owing to the enzymatic activity of thrombin [80]. Better knowledge regarding the interlinkage among structural properties and activity of heparin creates an opportunity for the development of new drugs having more specificity and improved anti-coagulating properties. Furthermore, this structural-activity connection is also helpful in exploring various biomedical applications like anti-viral, anti-inflammatory, anti-cancer, and wound healing properties [78,79,81,82]. DS and CS chains comprise of sulfated N-acetylgalactosamine and iduronic acid (in case of DS)/glucuronic acid (in case of CS) disaccharide repeating units [83,84]. Both of these are functionally characterized as bioactive polysaccharides owing to their presence as a vital molecule in the extracellular matrix of the brain, which helps in regulation of cell migration, adhesion, and proliferation while healing of wounds, as well as proper signaling of growth factor in the skeleton [85,86,87,88]. 

On the other hand, HA (hyaluronic acid) primarily isolated form animal tissues comprise of the linear structure of non-sulfated glycosaminoglycan molecules with repeated units of β-(1-3)-*N*-acetyl-d-glucosamine, and β-(1-4)-d-glucuronic acid. It is also a vital constituent of the brain’s extracellular matrix, which promotes mediation of cellular signaling, helps in healing of wounds, and morphogenesis. Owing to these properties, HA and their associated derivatives are predominantly being applied by the medical industry in eye surgery, viscosupplementation, and as a biomedical tool for drug delivery [89,90,91]. Chitin is known to be the most abundant polysaccharide present in the exoskeleton of shrimps and crabs, the cell walls of yeast and fungi, and the cuticles of insects. It is by far the second most copious polymer after cellulose and comprises of a random distribution of β-(1-4)-linked *N*-acetyl-glucosamine and *N*-glucosamine [92,93]. Industrially, chitosan is characterized as a vital derivative of chitin and is practically obtained due to partial deacetylation of chitin either by the action of enzyme chitin deacetylase or under alkaline conditions [94]. Bioactive properties (anti-cancer, anti-tumor, anti-bacterial, anti-inflammatory, etc.) associated with oligomers of chitosan and chitin, yielded owing to partial acidic hydrolysis, have earlier been documented [95]. 

## 3. Extraction and Quantification of Bioactive Polysaccharides

In general, bioactive potentials of naturally occurring polysaccharides are greatly dependent upon their molar mass and level and distribution of groups/side chains on the backbone. Therefore, isolation of polysaccharides from complex cellular plant matrices while keeping their bioactivity intact is of great significance. During the last decade, various innovative green extraction techniques (microwave-assisted, ultra-sonication, supercritical fluid extraction, and hot water extraction) are in practice for isolation of bioactive polysaccharides. These techniques have acquired great attention of scientists and researchers mainly due to their increased extraction rates, cost-effective nature, enviro-friendly characteristics, and structure preservative potentials [73,96,97].

Numerous scientific investigations have been implemented for the extraction and isolation of bioactive polysaccharides. The type of method adopted defines the physicochemical properties and antioxidant potential of isolated polysaccharides. For this purpose, a group of scientists compared the structural and antioxidant properties of bioactive polysaccharides extracted from Barrenwort (*Epimedium acuminatum*) through various extraction techniques (microwave-assisted, enzymatic, hot water extraction, and ultrasound-assisted extraction). They were of the view that bioactive polysaccharides isolated from hot water extraction had the highest antioxidant properties as compared to those extracted from other techniques, while their physicochemical properties were the same [98]. The hot water extraction method used in combination with various latest techniques (enzymatic pre-treatment, microwave, and ultrasound-assisted) are useful in increasing the yield and extraction productivity of polysaccharides. Likewise, enzymatic pre-treatment of raw material prior to extraction resulted in reduced extraction time, minimized the use of extraction solvents, preserved the bioactivities of the polysaccharides, and was energy efficient as compared to non-enzymatic pre-treated techniques [99]. In recent times, various ionic liquids have been formulated which aids in extraction of polysaccharides in a shorter time and at lower temperatures [100].

In addition, this purification of polysaccharides from the crude extract is really of great importance as the linkage among structure and safety of products formed for food, pharmaceutical, and biomedical application depends on this. Purification could be achieved by using various techniques (gel filtration, ion exchange and affinity chromatography, ethanolic precipitation, and fractional precipitation), individually or in combination [32]. 

## 4. Biomedical Applications

Polysaccharides and their derived compounds are medicinally more preferred as compared to synthetic polymers owing to their biodegradability, non-toxic nature, biocompatibility, and low processing expenses. Mentioned benefits related to polysaccharides isolated from natural sources make them a valuable ingredient in the fields of pharmaceuticals, nutraceuticals, food, and cosmetic industries. At the present time, polysaccharides are been used in healthcare and disease control, while various novel areas have also been discovered like in cancer diagnosis, inhibition, and treatment; in drug delivery; in anti-bacterial and anti-viral perspectives; and in tissue engineering [92,119]. Therefore, this segment highlights the use of bioactive polysaccharides against various metabolic syndromes and in the above-mentioned novel areas. 

### 4.1. Anti-Microbial and Antiviral

Various clinical investigations have authenticated that oral administration of pectin to infants and children significantly reduced diarrhea and other intestinal infections. This may be because of the decreased concentration of pathogenic bacteria like *Citrobacter*, *Salmonella*, *Enterobacter*, *Shigella*, *Proteus*, and *Klebsiella* [120]. A linear relationship has been documented among the concentration of probiotics and intestinal health [28].

The bioactive potential of fucoidans—a sulphated polysaccharide derived from marine brown seaweeds—have demonstrated noteworthy anti-viral potential against the cytomegalovirus, HIV, and HSV (herpes simplex virus) [121]. Additionally, few other seaweed-extracted polysaccharides like sulphated rhamnogalactans, carrageenans, and fucoidans have shown an inhibitory effect on viruses (HSV and HIV). Fucoidan comprises of a large quantity of L-fucose and sulphate groups along with fractions of galaturonic acid, xylose, mannose, and galactose. *Undaria pinnatifida* (marine brown alga) contains fucoidans and have been used in bone health supplementation mainly due to stimulation of osteoblastic cell differentiation. This sulphated polysaccharide has also been known to possess preventing action on UV-B-induced matrix metalloproteinase-1 (MMP-1) expression by inhibiting the ERK (extra-cellular signal regulated kinases) pathways. Therefore, it could be utilized as a functional ingredient in dermal ointments to prevent from skin photo-aging [40].

Some of the other fractions of algae have properties of virucidal and enzyme inhibitory activity inhibiting the formation of the syncytium. Besides, the sulfate group present is necessary for the anti-HIV activity and potency increases with the degree of sulfation. 

### 4.2. Anti-Tumor/Cancer

Numerous scientists have explored dietary fibers as possessing potent anti-cancer properties. Amongst all, pectin has been investigated to reduce cancerous cell migration and tumor growth in a rat model that were administrated with modified citrus pectin [122]. This may be due to binding of pectin to galectin-3, which results in inhibitory action on some of its functional activities [123]. Anti-tumor mode of actions associated with dietary pectin are related to their immune-potentiation, probiotic properties, tumor growth inhibition, anti-mutagenic potential, and regulatory action of transformation-related oncogenes [124,125]. Anti-tumor mechanisms associated with pectin could be due to cellular immunological potential [126].

According to a study, ginseng polysaccharides were found to have a stimulating effect on DCs (dendritic cells) causing an elevated formation of IFN-g (interferon-g) [127]. It has also been documented that acidic ginseng polysaccharides (GPs) enhanced the production of cytotoxic cells against tumors and promoted macrophages for the production of Th1 and Th2 (helper type 1 and 2) cytokines [128,129]. Depending upon disease environment or timing of treatments, ginseng polysaccharides extracted from *Panax ginseng* demonstrated immuno-modulating perspectives mainly in an immunosuppressing or immuno-stimulating manner [130]. Acidic GP also revealed modulating action on the concentrations of antioxidative enzymes like GPx (glutathione peroxidase) and SOD (superoxide dismutase) probably due to induction of regulating cytokines [131,132]. Likewise, Lemmon et al. [132] found that the immuno-stimulating potential of acidic GPs isolated from American ginseng (*Panax quinquefolius*) was actually mediated by polysaccharides having molecular weight more than 100 kDa [34].

Furthermore, scientists have proven the fact that heparin administration may also have a beneficial impact on cancer and inflammation. Anti-cancerous, anti-inflammatory, and anti-tumor properties associated with heparin and its low molecular weight species are owing to the pathological functions of heparan sulfate (HS) chains of proteoglycan structure (HSPGs). Outcomes of an investigation validated that heparin transfers GRs (growth factors) stored by HS chains of HSPGs in the ECM (extracellular matrix) and on cell surfaces. Full-size heparin has potent pro-angiogenic properties as it increases the production of ternary complexes of heparin bound FGF2 and VEGF with GF receptors [45].

### 4.3. Anti-Obesity and Hypocholesterolemia

Numerous trials have shown a direct relationship between consumption of dietary fiber, rich diet/dietary fiber supplementation, and weight loss [133,134,135,136,137]. According to a meta-analysis comprising of 22 clinical trials, it was documented that a 12 g increase in the content of daily fiber intake resulted in a 10% decrease in energy intake along with a 1900 g decline in body weight [138]. More precisely, the administration of glucomannan (1.24 g/day) along with energy-restricted diet for five consecutive weeks caused a significant decrease in body weight as compared to the placebo group [139].

In a clinical trial on healthy volunteers, a drink containing oat β-glucan (10.5 g/400 g and 2.5 and 5 g/300 g) enhanced fullness sensation as compared to fiber-free drink [140,141]. Likewise, in healthy adolescents subjected to biscuits enriched with barley β-glucan (5.2%) helped in suppressing appetite ratings as compared to control biscuits [142]. Similarly, administration of bread formulated by barley β-glucan (3%) to volunteers resulted in decrease of hunger and increased satiety and fullness. This also resulted in a noteworthy decrease in energy intake at successive lunches [143]. On the other hand, a bar prepared from barley β-glucan (1.2 g) subjected to healthy volunteers did not change scores for energy intake and appetite scores as compared to control bars [144]. Effects of β-glucan on satiety depends upon the concentration, molecular weight (31–3100 kDa), solubility, and food carrying it [38].

Furthermore, a group of scientists investigated the hypocholesterolemic perspectives of a dietary supplement comprising equal content of konjac glucomannan (KGM) and chitosan [145]. The concentration of serum total cholesterol and low-density lipoprotein cholesterol (LDL-c) significantly reduced at the end of the trial (28th day). Fecal excretion of bile acids and neutral sterol were observed more at the commencement of the study as compared to the initiation of the study. Similarly, Chen et al. [146] investigated the impact of KGM supplementation (3.6 g/day) on levels of glucose and lipid biomarkers in hypercholesterolemic type-2 diabetic patients. Twenty-two diabetic patients having increased serum cholesterol content were selected for this study. As compared to the placebo group, KGM supplemented group showed decreased levels of LDL-c (20.7%), fasting glucose (23.2%), serum cholesterol (11.1%), and Apo-B (12.9%). Fecal bile acid and neutral sterol content were elevated significantly by 75.4% and 18.0%, respectively. Results of all the mentioned trials revealed that KGM supplementation could assist in the treatment of hypercholesterolemic diabetic patients [44].

### 4.4. Anti-Diabetic

Scientific evidences have shown that β-glucan can contribute to control glycemic responses. Numerous factors are found to affect such interactions like the nature of the food, concentration, and molecular weight of β-glucan. Among all these, the dose of β-glucan is considered to be the most important factor in regulating the impact of fiber on glycemic responses. As compared to other fibers, a small dose of β-glucan is sufficient to reduce the insulin and postprandial glucose responses in type 2 diabetic [147,148], healthy [149,150], and hyperlipidemic subjects [151]. Studies have revealed that consumption of breakfasts comprising of 4, 6, and 8.6 g of β-glucan momentously reduced the mean concentration of serum insulin and glucose as compared to control non-insulin-dependent diabetic mellitus subjects [147]. The content of exogenous glucose was noticed as 18% less in a polenta meal containing oat β-glucan (5 g) as compared to a control polenta meal without oat β-glucan-subjected individuals [152]. Likewise, consumption of a meal consisting of 13C-labelled glucose and β-glucan (8.9 g), for a period of three days, reduced (21%) the levels of exogenous 13C-glucose in plasma as compared to control meal having no β-glucan [38,153]. 

### 4.5. Gastro-Protective

An experimental trial conducted by means of two diverse types of resistant starches (one a high amylose granular resistant corn starch and the other was high amylose non-granular, dispersed, and retrograded resistant corn starch) to evaluate the influence on blood lipid concentration, fecal SCFA and bulking, and glycemic indexes. This study also comprised of supplements containing low fiber control and high fiber control. Outcomes of this trial revealed that high fiber control (wheat bran) and both resistance starches subjected groups showed an elevation in the fecal bulk as compared to the low fiber control group. Likewise, the average ratio of fecal SCFAs and butyrate had progressive effects on colon health. Xanthan gum may also be used in milk as a prebiotic for lactic acid bacteria. Similar trials regarding prebiotics have demonstrated protective implications on the sustainability of cultures under the presence of bile salts and refrigeration and low pH conditions. According to a study, guar gum has the capability to change lipoprotein and postprandial lipid compositions. Supplementation of guar gum has an influence on lipoprotein composition, lipemia, and postprandial glycaemia [19].

Chen et al. [154] explored the effect of KGM supplementation on the gastrointestinal response in volunteer subjects. They were of the view that KGM supplementation significantly elevated the dry and wet stool weight and defecation frequency to 21.7%, 30.2%, and 27.0%, correspondingly. The improved dry fecal mass may be due to the existence of plant soluble materials. Nevertheless, the bacterial biomass of total bacteria, bifido-bacteria, and lactobacilli increased in fecal mass in KGM supplemented groups. Furthermore, reduction in fecal pH and elevation in fecal short chain fatty acids (SCFAs) resulted in increased colonic fermentation owing to KGM supplementation [44].

### 4.6. Immune Modulatory

Ginseng polysaccharides (GPs) have not only been known to possess immune-stimulating perspectives but also are found to suppress the proinflammatory responses. According to a recent study, novel neutral polysaccharide (PPQN) derived from an American ginseng root was documented to have a suppressing effect against inflammation. This activity was reported due to the inhibitory effect of isolated polysaccharide on inflammatory-related mediators such as cytokines (IL-1b, IL-6, TNF-a) and NO (nitric oxide) in comparison with LPS (lipopolysaccharide) treatment. Owing to this mode of action, novel neutral polysaccharide isolated from an American ginseng root could be used in modulating numerous inflammatory-related health implications (tumor, cancer, etc.) [155]. Similarly, another study reported the inhibitory influence of ginseng polysaccharides on immunological responses noticed in collagen-induced arthritic subject [156]. *P. quinquefolius* (American ginseng) is extensively used for the preparation of numerous herbal products. Extracts of *P. quinquefolius* were found to suppress the immune-inflammatory response, reduced the activity of neutrophils, induced the formation of cytokines in the spleen, and elevated the production of splenic-B lymphocytes and bone marrow [157,158,159,160].

*Platycodon grandifloras* is an herbaceous plant which is used as folk medicines since ancient times to curb various diseases like asthma, bronchitis, and pulmonary tuberculosis. Proximate composition of *P. grandifloras* reveals that it is a rich source of carbohydrates (90%), protein (2.4%), ash (1.5%), and fat (0.1%). Polysaccharides extracted from roots of *P. grandifloras* have been reported to possess antidiabetic, hypolipidemic and hypocholesterolemic properties [161]. Furthermore, the inulin-type polysaccharides isolated from *P. grandifloras* (PGs) roots validated the immune-modulating impact on macrophages and B-cells, but had no effect on T-cells [162]. 

### 4.7. Anti-Inflammatory

*Astragalus* polysaccharides (APS) are known to possess anti-inflammatory effects on cytokines of CD4^+^ T_h_ (T-helper) cells. In in-vitro antidiabetic models, an *Astragalus* polysaccharide has potentiated the lowering effect on the expression of T-helper 1 (Th1) and regulated the imbalance of Th1 and Th2. APS has reported to significantly enhance the gene expression of peroxisome-proliferator-activated receptor gamma (PPAR-γ) in a concentration-time dependent manner [163] and stimulated superoxide dismutase (SOD) anti-oxidative mechanism in type-1 diabetes mellitus (DM) models [164,165]. Moreover, APS reduced the expression of iNOS (inducible nitric oxide synthase) [122]. These inflammatory markers (NO, PPAR-γ, SOD, and iNOS) amongst diverse roles also perform numerous functions in regulating and stimulating inflammatory response [42]. 

Water-soluble sulfated polysaccharides (WSSPs) isolated from marine algae are also classified as anti-inflammatory compounds. On the other hand, very few pieces of evidence are present regarding anti-inflammatory perspectives of seaweed based sulfated polysaccharides. In vitro and in vivo studies have revealed that *Gracilaria verrucose-* and *Porphyra yezoensis*-derived sulfated polysaccharides stimulated the respiratory burst and phagocytosis in experimented mouse macrophages [40]. Orally administrated chondroitin sulfate (CS) isolated from cartilage of Skate (*Raja kenojei*) affected arthritic conditions in a dose-dependent manner in chondroitin sulfate-treated groups. Pre- and post-treated groups that were subjected to CS (1000 mg kg^−1^) revealed momentously decreased clinical scores as compared to vehicle treated groups. CS administration decreased the infiltration of inflammatory cells and prohibited from paw and knee joint destruction. Moreover, the results of RT-PCR showed that CS ingestion significantly repressed the expression of IL-1b (interleukin-1b), IFN-c (interferon-c), and TNF-α as compared to vehicle administrated group. The CS-treated group reduced the formation of rheumatoid arthritis responses (IgG and IgM) in collagen-induced arthritic mice (CIA) model. Outcomes of this study authenticate the shielding potential of chondroitin sulfate in CIA mice mainly due to the inhibitory effect of pro-inflammatory cytokines formation [43].

### 4.8. Neuro-Protective

*Acanthopanax senticosus* derived polysaccharides comprised of uronic acid (22.5%), proteins (18.7%), and carbohydrates (58.3%). It could be established that *Acanthopanax*-based polysaccharides may not only help in improving symptoms regarding nervous defects but also reduced the infarct volume and water content of the brain in rats having cerebral ischemia–reperfusion injury. Additionally, polysaccharides isolated from *A. senticosus* elevated SOD, IL-10, and GSH-Px concentration and reduces the levels of TNF-α, IL-1, and MDA in brains tissues of experimented rats. Conclusively, bioactive polysaccharides extracted from *A. senticosus* protected brain damage due to antioxidative potential and inhibitory action on stimulation of inflammatory cytokines [47]. 

### 4.9. Anti-Oxidant

Bioactive acidic polysaccharides extracted from *Polygonum multiflorum* showed significant antioxidative properties (hydroxyl peroxide, superoxide anion radical, and hydroxyl radical), protein glycation and lipid oxidation. In addition to this, the intraperitoneal (i.p.) administration of *P. multiflorium*-based polysaccharides may increase the serum concentration of antioxidative characteristics in cyclophosphamide-induced anemic mice. Results of this study validate the use of *P. multiflorium* as a novel antioxidant tool to prevent oxidation [41]. Sulfated polysaccharides not only act as dietary fiber but also act as a natural antioxidant agent. They are responsible for the antioxidant properties possessed by marine algae. Various studies have recognized the use of numerous classes of SPs (alginic acid, Fucoidan, and laminaran) as potent antioxidative agents. Antioxidative potential of SPs has classified by multiple in-vitro methods such as DPPH, FRAP, NO, ABTS radical scavenging, superoxide radical scavenging assay, and the hydroxyl radical scavenging assay. Additionally, Xue et al. [166] stated that many marine-based sulfated polysaccharides have shown antioxidant potential in organic solvents and a phosphatidylcholine-liposomal suspension [40].

### 4.10. Tissue Engineering

Application of bioactive polysaccharides and their derivatives in the field of tissue engineering (cell differentiation, cell adhesion, cell remodeling, cell proliferation, and cell responsive degradation) has opened new horizons in medical research, and therefore impelled the researchers to regenerate new tissues and define the structure of cellular growth [92]. Various bioactive polysaccharides including starch, chitosan, chondroitin sulfate, alginate, cellulose, chitin, hyaluronic acid, and their derivatives are being used as biomaterials in applications for tissue engineering [167]. Application of these bioactive polysaccharides as scaffolds in tissue engineering are required to accomplish some requirements such as non-toxicity, biodegradability having controlled the rate of degradation, biocompatibility, structural integrity, and suitable porosity [92].

Chitosan and chitin have all the required potential to act as scaffolds for tissue engineering mainly due to their mechanical strength, degradability, and immunogenicity. Hence, for tissue engineering they are being developed as 3D-hydrogels, free standing films, porous sponges, and fibrous scaffolds, inside which for in-vitro/in-vivo cultures the most suitable cell types are needed [168]. Designing of 3D-chitin/chitosan-based hydrogels and sponge scaffolds, and 2D-scaffolds for the purpose of cartilage and tendon regenerations, for encapsulation of stem cells ensuring their therapeutic application, and for utilizing these as a tool for regenerative medicine have been reported in numerous researches [169,170]. Furthermore, for bone regeneration purpose, the tissue engineering industry has formulated combinations of chitosan and hydroxyapatite and grafted chitosan and carbon nanotubes [171]. Along with this, numerous other bioactive polysaccharides like cellulose, hyaluronic acid, and starch have also been studied in detail to validate their use as a biomaterial for skin, bone, and cartilage tissue engineering [95].

### 4.11. Wound Healing and Wound Dressing

Numerous bioactive polysaccharides (alginate, chitin, hyaluronan, chitosan, and cellulose) are used for the preparation of wound healing materials owing to their intrinsic bio-compatible, less toxic, and pharmaceutical activities [172,173]. For instance, hyaluronan is a vital extracellular component possessing distinctive viscoelastic, hygroscopic, and rheological characteristics are well known for its tissue repairing properties owing to their physicochemical potentials and specific interaction with cells and extracellular matrices. It is documented earlier that hyaluronan has a multidimensional role regarding the repairing process of cell or wound healing specifically inflammation, granulation, formation of tissues, re-epithelialization, and remodeling. Various hyaluronan-derived products like esterified, cross-linked, or chemically modified products are medicinally used for wound healing and tissue repairing purposes [174]. While designing bioactive material for tissue engineering their wound healing properties is of great interest.

Naturally available wound dressing films either prepared by encapsulation or simply dispersion of the sodium alginate matrix in essential oils from cinnamon, lemon, tea, lemongrass, lavender, elicriso italic, peppermint, chamomile blue, and eucalyptus have demonstrated exceptional anti-fungal and anti-microbial activities, and therefore their application in disposable dressings for wounds could also be found [175]. Development of wound dressings obtained from cross-linkage between chitosan/silk fibroin blending membranes and di-aldehyde alginate have found to enhance cellular proliferative properties, suggesting their applications as wound healing agents [176]. Preparation of freestanding sodium alginate films or Ca^2þ^ cross-linked alginate beads was achieved by mixing aqueous dispersions of PVPI (povidone iodine) and Na-Alg. These films/beads showed anti-fungal/anti-bacterial properties along with control release of povidone iodine into wounds as these products came into direct contact with the moist environment [175]. These applications validate the use of these products therapeutically in wound dressings. Some innovative wound dressings were prepared for external treatment of wounds by in situ injection of nanocomposite hydrogels that actually comprised of oxidized alginate, curcumin, and N, O-carboxymethyl chitosan. Results of various in vitro, in vivo, and histological investigations have proven the use of nanocurcumin, N, O-carboxymethyl chitosan, and oxidized alginate-based hydrogels as novel tools in wound dressings for their application as wound repairing agents. Furthermore, gamma radiations were successfully employed for the synthesis of silver nanoparticles comprising of alginate and polyvinyl pyrrolidone (PvP)-based hydrogels. These products have scientifically shown their capability regarding the prevention of fluid accumulations in exudate wounds [177]. The amalgamation of nano-silver particles provides a promising anti-microbial property and hence made these PvP-alginate hydrogels most appropriate for wound healing and dressing. Other than alginate and their associated derivatives, numerous other naturally occurring polysaccharides like hyaluronic acid, cellulose, chitosan, and chitin have been investigated by researchers to assess their wound healing applications [178]. 

### 4.12. Drug Delivery and Controlled Release

Application of bioactive polysaccharides as a novel agent in drug delivery and controlled release has also been studied by scientists owing to their least toxicity, minimum immunogenicity, and biocompatibility. Various naturally occurring polysaccharide-based drug delivery systems are in practice due to their targeted delivery/controlled release, shielding effect against premature degradation of drugs, improvement of intracellular transportation, enhancement of bioavailability of drugs, as well as delivery of small interfering RNA, antigens, and genes [179]. Delivery systems mentioned here usually possess covalent/ionic cross-linkages, poly-electrolyte complexes, conjugates of polysaccharides and drugs, and self-assembly [179]. Release of 3-D cross-linked drugs could be triggered by varying redox potential, pH, light, ions, temperature, and application of magnetic and/or electric fields [180]. Mainly the three most abundantly used polysaccharides i.e., alginate, chitin, cellulose, and chitosan are overviewed in detail as under in this portion.

Pharmaceutic application of cellulose and their associated derivatives could be classified either as pharmaceutical excipients for protecting purposes or as bioactive molecules themselves. Application of bioactive polysaccharides as pharmaceutical excipients in orally administrated drug delivery systems have been explored to enhance the solubility and bioavailability of drugs, to increase the final product (drug) stability, and to attain release profile from final formulations [181]. These days, microcrystalline cellulose, rice, and corn starches have been broadly engaged in formulations of capsule diluents, tablet dis-integrants, and glidants. Various cellulose derivatives like HPMC (hydroxypropyl methyl cellulose), MC (methyl cellulose), HPC (hydroxypropyl cellulose), and HEC (hydroxyethyl cellulose) possessing better physiochemical properties as compared to cellulose are evidently being used in pharmaceutical industries [182]. For instance, HPMC phthalate has significant pH depending solubility, specifically, stability under acidic conditions of the stomach while soluble in mild acidic to slight alkaline solutions and, hence, are being applied for controlled release of intestinal targeted drugs. In recent times, nanocellulose-based drug delivery systems comprising of CNCs (cellulose nanocrystals), NFC (nanofibrillated cellulose), and BC (bacterial cellulose) have been investigated comprehensively [183]. For example, the binding and release of the hydrochloride salt of doxorubicin and tetracycline have been explored extensively due to ionic cross-linked systems, in which sulfate groups on cellulose nanocrystals possessing negative charge are reversibly cross-linked ionically to counterpart positively charged drugs. Likewise, nanofibrillated cellulose-based films have also been investigated for entrapment of drugs and are being used in pharmaceutical industries for the production of long-lasting drug release systems [184]. 

Reconnoitering the application of chitin/chitosan as bio-molecular delivery vectors have impelled the scientists for the development of therapeutic drug delivery systems like siRNA (small interfering RNA) carriers, antigens, and genes [185]. In vivo, therapeutic application of chitosan-based siRNA carries has shown great potential as a tool for gene expression associated diseases. Inhibitory influence on human colorectal cancer gene expression due to the application of chitosan-siRNA nanoparticles have been studied in an earlier study [186]. It was noticed that chitosan-siRNA nanoparticles developed by ionic gelation with Na-tri-polyphosphate demonstrated a more targeted dene inhibiting impact owing to increased binding and loading effectiveness. Long-lasting delivery of encapsulated antigens or intra-dermal vaccines administrated through chitosan microneedles transdermal delivery systems are documented to deliver more sustainable immune stimulation [187]. Though, the sensitivity of pH could also affect the stability issues of the drug delivery systems [179]. Various other bioactive polysaccharides like chondroitin, pectin, xanthan gum, dextran, chitin, gellan gum, chitosan, and dextran are also being used for controlled drug delivery [1,181].

## 5. Conclusions

Bioactive polysaccharides have acquired significant attention from scientists as functional biomolecules for the development of innovative and value-added products in the fields of pharmaceutics, food, cosmetics, and the biomedical industry. Their therapeutic application is mainly due to their bio-degradable, non-toxic, and bio-compatible nature. Extraction and isolation of naturally occurring bioactive polysaccharides possessing high purity with maximum extraction yield, meanwhile keeping in view that the native structure remains intact, are of great future concern and remains a field for further exploration. Momentous results to authenticate the use of these polysaccharides as a novel tool in the pharmaceutical and medicinal industry will require a multidimensional approach from scientists of various fields like healthcare, food science, organic chemistry, material science and engineering, as well as plant biology. 

## Figures and Tables

**Table 1:** Biological activities of bioactive polysaccharides.

Polysaccharides	Biological Activities	Mechanism of Action	References
β-glucan	Anti-obesity activity	Reduced energy intake Increase in fullness and satiety Decreased hunger and increased satiety and fullness.	[38]
Pectin, Algal Fucoidan	Anti-microbial activity	Inhibitory effects on the entry of enveloped viruses including herpes and HIV into cells Inhibit the formation of syncytium formation	[39,40]
Fucoidan, Polygonum multiflorum Thunb Polysaccharides	Anti-oxidant activity	Inhibited ERK and p38-MAPK signaling pathways scavenging free radicals (e.g., superoxide anion radical, hydroxyl radical, and hydroxyl peroxide), Prevents lipid oxidation and protein glycation Inhibits the formation of ROS and RNS	[40,41]
Red algae sulfated polysaccharides, Carrageenan, Fucoidan, Chondroitin sulfate	Anti-inflammatory activity	Lowered the expression of inducible nitric oxide synthase (iNOS) Inhibited the expressions of TNF-a, interleukin-1b (IL-1b) and interferon-c (IFN-c) Repressed pro-inflammatory cytokines Suppresses the activity of COX-2	[40,42,43]
β-glucan	Anti-diabetic activity	Suppressed the formation of AGE Reduced postprandial glucose and insulin responses Increases the level of antioxidant enzymes in the body	[38]
Konjac glucomannan	Hypo-cholesterolemic activity	Reduced the plasma cholesterol Significantly lowered Serum total, HDL-C, and LDL-C Reduces the concentration of serum MDA	[44]
Pectin, Ginseng polysaccharides, Heparan sulfate	Anti-tumour activity	Constrains the production of prostaglandin E2 Prevents from the oxidation of DNA Stimulates macrophages to produce helper types 1 and 2 (Th1 and Th2) cytokines Promotes the formation of ternary complexes Displaces growth factors	[39,45,46]
Ginseng polysaccharides	Immune modulatory activity	Downregulates the secretion of inflammation related mediator nitric oxide (NO) and cytokines (TNF-a, IL-6, and IL-1b) Reduces the activation of neutrophils	[45]
β-glucan, Pectin, Gums, Konjac glucomannan	Gastro-protective activity	Supplement increased faecal bulk Alter postprandial lipid and lipoprotein composition	[19,44]
Acanthopanax polysaccharides	Neuro-protective activity	Increase SOD and GSH-Px activities and IL-10 levels Reduces the levels of MDA, IL-1, and TNF-α Prevents the formation of inflammatory cytokines	[47]

**Table 2:** Extraction techniques with reference to specific polysaccharides.

Type	Name	Sources	Extraction Techniques	Quantity/Yield	References
Dietary fibres	Cellulose	Coconut fibre	Acid Hydrolysis	32.8%	[101]
			Ammonium persulphate oxidation	49.6%	
			Ultrasound extraction	88.1%	
	Hemicelluloses	Wheat bran	Alkaline extraction	33.32%–64.1%	[102]
			Hydrothermal extraction	9% (14)	
	Pectins	Orange peels	Microwave-assisted extraction	24.2%	[103]
			Conventional extraction	18.32%	
			Ultrasound extraction	19.24%	
	B-glucan	Barley	Acidic extraction	4.6%	[104]
		Oats		6.97%	
		Barley bran	Alkaline extraction	5.6%–11.9%	
		Oat bran		3.9%–8.0%	
		Wheat bran		2.15%–2.51%	
		Barley		3.94%	
		Barley	Water extraction	2.5%–5.4%	
		Oat		2.1%–3.9%	
		Barley	Enzymatic extraction	5.22%	
		Oat		13.9%	
	Gums	Durian fruit	Aqueous extraction	59.7%	[105]
	Konjac glucomannan	*Amorphophallus konjac* plant	Water extraction using Al2(SO_4_)_3_	59.02%	[106]
			Ethanol extraction	65.23%	
Herbs	Ginseng polysaccharides	*Panax ginseng* roots	Subcritical water extraction	63.1%	[107]
			Water extraction	14.71%	
			Ethanol extraction	17.96%	
	*Astragalus* polysaccharides	*Astragalus* roots	Water extraction	11.6% (20)	[108]
			Microwave-assisted extraction	16.07%	
			Ultrasound-assisted extraction	24.12%	
			Enzymatic hydrolysis extraction	9.78%	
	*Polygonum multiflorum* Thunb Polysaccharides	*Polygonum multiflorum* Thunb root	Ultrasound-assisted extraction	2.90%–4.72% (21)	[109]
			Ethanolic extraction	4.9% (22)	[110]
Algae and lichens	Green algae sulfated polysaccharides	Green algae, *Caulerpa racemosa*	Soxhlet extraction	10%–20% (23)	[111]
			Ultrasound assisted extraction	8.3% (24)	[112]
	Red algae sulfated polysaccharides (porphyran)	Red algae, *Porphyra haitanensis*	Ultrasound-assisted extraction	6.24% (25)	[113]
	Green algae sulfated Rhamnan	Green algae, *Monostroma latissimum*	Hot water extraction	15.9%	[114]
			Microwave-assisted extraction	53.1% (26)	
	Algal Fucoidan	Brown algae, *Ecklonia cava*	Aqueous extraction	53.33%	[115]
			Enzymatic extraction	57.00% (27)	
			Microwave assisted extraction	18.22% (30)	[116]
β-Glucans lichenan	Lichenan	*Cetraria islandica*	Hot water extraction	50.9%	[117]
	Pustulan	*Lasallia pustulata*	Hot water extraction	38%	
Fungi	Monosaccharides and polysaccharides	*Cordyceps militaris*, *Dictyophora* spp.	Hot water, alkali, and acidic solutions	6.36%–24.30%	[56,118]

## References

[1] Zhang Y., Wang F. (2015). Carbohydrate drugs: Current status and development prospect. Drug Discov. Ther..

[2] Li P., Wang F. (2015). Polysaccharides: Candidates of promising vaccine adjuvants. Drug Discov. Ther..

[3] Do Amaral A.E., Petkowicz C.L.O., Mercê A.L.R., Iacomini M., Martinez G.R., Rocha M.E.M., Cadena S.M.S.C., Noleto G.R. (2015). Leishmanicidal activity of polysaccharides and their oxovanadium (iv/v) complexes. Eur. J. Med. Chem..

[4] Zong A., Cao H., Wang F. (2012). Anticancer polysaccharides from natural resources: A review of recent research. Carbohydr. Polym..

[5] Colegate S.M., Molyneux R.J. (2007). Bioactive Natural Products: Detection, Isolation, and Structural Determination.

[6] Zhang C., Gao Z., Hu C., Zhang J., Sun X., Rong C., Jia L. (2017). Antioxidant, antibacterial and anti-aging activities of intracellular zinc polysaccharides from grifola frondosa sh-05. Int. J. Biol. Macromol..

[7] Sinha V., Kumria R. (2001). Polysaccharides in colon-specific drug delivery. Int. J. Pharm..

[8] Dong B., Hadinoto K. (2017). Direct comparison between millifluidic and bulk-mixing platform in the synthesis of amorphous drug-polysaccharide nanoparticle complex. Int. J. Pharm..

[9] Jung B., Shim M.-K., Park M.-J., Jang E.H., Yoon H.Y., Kim K., Kim J.-H. (2017). Hydrophobically modified polysaccharide-based on polysialic acid nanoparticles as carriers for anticancer drugs. Int. J. Pharm..

[10] Nuti E., Santamaria S., Casalini F., Yamamoto K., Marinelli L., La Pietra V., Novellino E., Orlandini E., Nencetti S., Marini A.M. (2013). Arylsulfonamide inhibitors of aggrecanases as potential therapeutic agents for osteoarthritis: Synthesis and biological evaluation. Eur. J. Med. Chem..

[11] Chen Q., Mei X., Han G., Ling P., Guo B., Guo Y., Shao H., Wang G., Cui Z., Bai Y. (2015). Xanthan gum protects rabbit articular chondrocytes against sodium nitroprusside-induced apoptosis in vitro. Carbohydr. Polym..

[12] An H.J., Lebrilla C.B. (2011). Structure elucidation of native n-and o-linked glycans by tandem mass spectrometry (tutorial). Mass Spectrom. Rev..

[13] MaKi-Arvela P.I., Salmi T., Holmbom B., Willfor S., Murzin D.Y. (2011). Synthesis of sugars by hydrolysis of hemicelluloses-a review. Chem. Rev..

[14] Yang L., Zhang L.-M. (2009). Chemical structural and chain conformational characterization of some bioactive polysaccharides isolated from natural sources. Carbohydr. Polym..

[15] Xiao Z., Tappen B.R., Ly M., Zhao W., Canova L.P., Guan H., Linhardt R.J. (2010). Heparin mapping using heparin lyases and the generation of a novel low molecular weight heparin. J. Med. Chem..

[16] Gatti G., Casu B., Hamer G., Perlin A. (1979). Studies on the conformation of heparin by 1h and 13c nmr spectroscopy. Macromolecules.

[17] Varki A., Cummings R., Esko J., Stanley P., Hart G., Aebi M., Darvill A., Kinoshita T., Packer N., Prestegard J. (2017). Oligosaccharides and Polysaccharides—Essentials of Glycobiology.

[18] Pool-Zobel B.L. (2005). Inulin-type fructans and reduction in colon cancer risk: Review of experimental and human data. Br. J. Nutr..

[19] Chawla R., Patil G. (2010). Soluble dietary fiber. Compr. Rev. Food Sci. Food Saf..

[20] Tungland B., Meyer D. (2002). Nondigestible oligo-and polysaccharides (dietary fiber): Their physiology and role in human health and food. Compr. Rev. Food Sci. Food Saf..

[21] Weng L.-C., Lee N.-J., Yeh W.-T., Ho L.-T., Pan W.-H. (2012). Lower intake of magnesium and dietary fiber increases the incidence of type 2 diabetes in taiwanese. J. Formos. Med. Assoc..

[22] Casiglia E., Tikhonoff V., Caffi S., Boschetti G., Grasselli C., Saugo M., Giordano N., Rapisarda V., Spinella P., Palatini P. (2013). High dietary fiber intake prevents stroke at a population level. Clin. Nutr..

[23] Viuda-Martos M., López-Marcos M., Fernández-López J., Sendra E., López-Vargas J., Pérez-Álvarez J. (2010). Role of fiber in cardiovascular diseases: A review. Food Sci. Food Saf..

[24] Whelton S.P., Hyre A.D., Pedersen B., Yi Y., Whelton P.K., He J. (2005). Effect of dietary fiber intake on blood pressure: A meta-analysis of randomized, controlled clinical trials. LWW.

[25] Chau C.-F., Huang Y.-L., Lin C.-Y. (2004). Investigation of the cholesterol-lowering action of insoluble fibre derived from the peel of citrus sinensis l. Cv. Liucheng. Food Chem..

[26] Kendall C.W., Esfahani A., Jenkins D.J. (2010). The link between dietary fibre and human health. Food Hydrocoll..

[27] Lunn J., Buttriss J. (2007). Carbohydrates and dietary fibre. Nutr. Bull..

[28] Anderson J.W., Baird P., Davis R.H., Ferreri S., Knudtson M., Koraym A., Waters V., Williams C.L. (2009). Health benefits of dietary fiber. Nutr. Rev..

[29] Brown L., Rosner B., Willett W.W., Sacks F.M. (1999). Cholesterol-lowering effects of dietary fiber: A meta-analysis. Am. J. Clin. Nutr..

[30] Tang W., Hemm I., Bertram B. (2003). Recent development of antitumor agents from chinese herbal medicines. Part ii. High molecular compounds. Planta Med..

[31] Harlev E., Nevo E., Lansky E.P., Ofir R., Bishayee A. (2012). Anticancer potential of aloes: Antioxidant, antiproliferative, and immunostimulatory attributes. Planta Med..

[32] Thakur M., Weng A., Fuchs H., Sharma V., Bhargava C.S., Chauhan N.S., Dixit V.K., Bhargava S. (2012). Rasayana properties of ayurvedic herbs: Are polysaccharides a major contributor. Carbohydr. Polym..

[33] Tian L., Zhao Y., Guo C., Yang X. (2011). A comparative study on the antioxidant activities of an acidic polysaccharide and various solvent extracts derived from herbal houttuynia cordata. Carbohydr. Polym..

[34] Jin M., Huang Q., Zhao K., Shang P. (2013). Biological activities and potential health benefit effects of polysaccharides isolated from lycium barbarum l.. Int. J. Biol. Macromol..

[35] Li T., Peng T. (2013). Traditional chinese herbal medicine as a source of molecules with antiviral activity. Antivir. Res..

[36] Harhaji T.L.M., Mijatović S.A., Maksimović-Ivanić D.D., Stojanović I.D., Momčilović M.B., Tufegdžić S.J., Maksimović V.M., Marjanovi Ž.S., Stošić-Grujičić S.D. (2009). Anticancer properties of ganoderma lucidum methanol extracts in vitro and in vivo. Nutr. Cancer.

[37] Ke M., Zhang X.-J., Han Z.-H., Yu H.-Y., Lin Y., Zhang W.-G., Sun F.-H., Wang T.-J. (2011). Extraction, purification of lycium barbarum polysaccharides and bioactivity of purified fraction. Carbohydr. Polym..

[38] El Khoury D., Cuda C., Luhovyy B., Anderson G. (2011). Beta glucan: Health benefits in obesity and metabolic syndrome. J. Nutr. Metab..

[39] Zhang W., Xu P., Zhang H. (2015). Pectin in cancer therapy: A review. Trends Food Sci. Technol..

[40] Wijesekara I., Pangestuti R., Kim S.-K. (2011). Biological activities and potential health benefits of sulfated polysaccharides derived from marine algae. Carbohydr. Polym..

[41] Zhu W., Xue X., Zhang Z. (2017). Structural, physicochemical, antioxidant and antitumor property of an acidic polysaccharide from polygonum multiflorum. Int. J. Biol. Macromol..

[42] Agyemang K., Han L., Liu E., Zhang Y., Wang T., Gao X. (2013). Recent advances in astragalus membranaceus anti-diabetic research: Pharmacological effects of its phytochemical constituents. Evid.-Based Complement. Altern. Med..

[43] Volpi N. (2011). Anti-inflammatory activity of chondroitin sulphate: New functions from an old natural macromolecule. Inflammopharmacology.

[44] Behera S.S., Ray R.C. (2016). Konjac glucomannan, a promising polysaccharide of amorphophallus konjac k. Koch in health care. Int. J. Biol. Macromol..

[45] Loh S.H., Park J.-Y., Cho E.H., Nah S.-Y., Kang Y.-S. (2017). Animal lectins: Potential receptors for ginseng polysaccharides. J. Ginseng Res..

[46] Casu B., Naggi A., Torri G. (2010). Heparin-derived heparan sulfate mimics to modulate heparan sulfate-protein interaction in inflammation and cancer. Matrix Biol..

[47] Xie Y., Zhang B., Zhang Y. (2015). Protective effects of acanthopanax polysaccharides on cerebral ischemia–reperfusion injury and its mechanisms. Int. J. Biol. Macromol..

[48] Kim S.-K., Li Y.-X. (2011). Medicinal benefits of sulfated polysaccharides from sea vegetables. Adv. Food Nutr. Res..

[49] Olafsdottir E.S., Ingólfsdottir K. (2001). Polysaccharides from lichens: Structural characteristics and biological activity. Planta Medica.

[50] Chattopadhyay N., Ghosh T., Sinha S., Chattopadhyay K., Karmakar P., Ray B. (2010). Polysaccharides from turbinaria conoides: Structural features and antioxidant capacity. Food Chem..

[51] Schepetkin I.A., Quinn M.T. (2006). Botanical polysaccharides: Macrophage immunomodulation and therapeutic potential. Int. Immunopharmacol..

[52] Omarsdottir S., Freysdottir J., Olafsdottir E.S. (2007). Immunomodulating polysaccharides from the lichen thamnolia vermicularis var. Subuliformis. Phytomedicine.

[53] Zambare V.P., Christopher L.P. (2012). Biopharmaceutical potential of lichens. Pharm. Biol..

[54] Martinichen-Herrero J., Carbonero E., Sassaki G., Gorin P., Iacomini M. (2005). Anticoagulant and antithrombotic activities of a chemically sulfated galactoglucomannan obtained from the lichen cladoniaibitipocae. Int. J. Biol. Macromol..

[55] Ngo D.-H., Kim S.-K. (2013). Sulfated polysaccharides as bioactive agents from marine algae. Int. J. Biol. Macromol..

[56] Zhang J., Wen C., Duan Y., Zhang H., Ma H. (2019). Advance in cordyceps militaris (linn) link polysaccharides: Isolation, structure, and bioactivities: A review. Int. J. Biol. Macromol..

[57] Meng X., Liang H., Luo L. (2016). Antitumor polysaccharides from mushrooms: A review on the structural characteristics, antitumor mechanisms and immunomodulating activities. Carbohydr. Res..

[58] Yu Y., Shen M., Song Q., Xie J. (2018). Biological activities and pharmaceutical applications of polysaccharide from natural resources: A review. Carbohydr. Polym..

[59] Negre-Salvayre A., Coatrieux C., Ingueneau C., Salvayre R. (2008). Advanced lipid peroxidation end products in oxidative damage to proteins. Potential role in diseases and therapeutic prospects for the inhibitors. Br. J. Pharmacol..

[60] Kamerling J.P., Gerwig G.J. (2007). Strategies for the structural analysis of carbohydrates. Compr. Glycosci..

[61] Feng F., Zhou Q., Yang Y., Zhao F., Du R., Han Y., Xiao H., Zhou Z. (2018). Characterization of highly branched dextran produced by leuconostoc citreum b-2 from pineapple fermented product. Int. J. Biol. Macromol..

[62] Chen L., Ge M.-D., Zhu Y.-J., Song Y., Cheung P.C.K., Zhang B.-B., Liu L.-M. (2019). Structure, bioactivity and applications of natural hyperbranched polysaccharides. Carbohydr. Polym..

[63] Cerning J. (1990). Exocellular polysaccharides produced by lactic acid bacteria. FEMS Microbiol. Rev..

[64] Pereira S., Micheletti E., Zille A., Santos A., Moradas-Ferreira P., Tamagnini P., De Philippis R. (2011). Using extracellular polymeric substances (eps)-producing cyanobacteria for the bioremediation of heavy metals: Do cations compete for the eps functional groups and also accumulate inside the cell?. Microbiology.

[65] Guezennec J. (2002). Deep-sea hydrothermal vents: A new source of innovative bacterial exopolysaccharides of biotechnological interest?. J. Ind. Microbiol. Biotechnol..

[66] Senni K., Pereira J., Gueniche F., Delbarre-Ladrat C., Sinquin C., Ratiskol J., Godeau G., Fischer A.-M., Helley D., Colliec-Jouault S. (2011). Marine polysaccharides: A source of bioactive molecules for cell therapy and tissue engineering. Mar. Drugs.

[67] Roger O., Kervarec N., Ratiskol J., Colliec-Jouault S., Chevolot L. (2004). Structural studies of the main exopolysaccharide produced by the deep-sea bacterium alteromonas infernus. Carbohydr. Res..

[68] Zhou Y., Cui Y., Qu X. (2019). Exopolysaccharides of lactic acid bacteria: Structure, bioactivity and associations: A review. Carbohydr. Polym..

[69] Kay E., Cuccui J., Wren B.W. (2019). Recent advances in the production of recombinant glycoconjugate vaccines. NPJ Vaccines.

[70] Gatenholm P., Tenkanen M. (2004). Industrially Isolated Hemicellulose.

[71] Barsett H., Ebringerová A., Harding S., Heinze T., Hromádková Z., Muzzarelli C., Muzzraelli R., Paulsen B., Elseoud O. (2005). Polysaccharides I: Structure, Characterisation and Use.

[72] Ebringerová A., Hromádková Z., Hříbalová V., Xu C., Holmbom B., Sundberg A., Willför S. (2008). Norway spruce galactoglucomannans exhibiting immunomodulating and radical-scavenging activities. Int. J. Biol. Macromol..

[73] Le Normand M., Mélida H., Holmbom B., Michaelsen T.E., Inngjerdingen M., Bulone V., Paulsen B.S., Ek M. (2014). Hot-water extracts from the inner bark of norway spruce with immunomodulating activities. Carbohydr. Polym..

[74] Aachary A.A., Prapulla S.G. (2011). Xylooligosaccharides (xos) as an emerging prebiotic: Microbial synthesis, utilization, structural characterization, bioactive properties, and applications. Food Sci. Food Saf..

[75] Van Ophoven A., Vonde K., Koch W., Auerbach G., Maag K.P. (2019). Efficacy of pentosan polysulfate for the treatment of interstitial cystitis/bladder pain syndrome: Results of a systematic review of randomized controlled trials. Curr. Med. Res. Opin..

[76] Li J., Mei X. (2006). Applications of Cellulose and Cellulose Derivatives in Immediate Release Solid Dosage.

[77] Chappell E.P., Liu J. (2013). Use of biosynthetic enzymes in heparin and heparan sulfate synthesis. Bioorg. Med. Chem..

[78] Linhardt R.J. (2003). 2003 Claude S. Hudson award address in carbohydrate chemistry. Heparin: Structure and activity. J. Med. Chem..

[79] Sakiyama-Elbert S.E. (2014). Incorporation of heparin into biomaterials. Acta Biomater..

[80] Schedin-Weiss S., Richard B., Hjelm R., Olson S.T. (2008). Antiangiogenic forms of antithrombin specifically bind to the anticoagulant heparin sequence. Biochemistry.

[81] Rajangam K., Behanna H.A., Hui M.J., Han X., Hulvat J.F., Lomasney J.W., Stupp S.I. (2006). Heparin binding nanostructures to promote growth of blood vessels. Nano Lett..

[82] Zhang F., Walcott B., Zhou D., Gustchina A., Lasanajak Y., Smith D.F., Ferreira R.S., Correia M.T.S., Paiva P.M., Bovin N.V. (2013). Structural studies of the interaction of crataeva tapia bark protein with heparin and other glycosaminoglycans. Biochemistry.

[83] Silbert J.E., Sugumaran G. (2002). Biosynthesis of chondroitin/dermatan sulfate. IUBMB Life.

[84] Takegawa Y., Araki K., Fujitani N., Furukawa J.-I., Sugiyama H., Sakai H., Shinohara Y. (2011). Simultaneous analysis of heparan sulfate, chondroitin/dermatan sulfates, and hyaluronan disaccharides by glycoblotting-assisted sample preparation followed by single-step zwitter-ionic-hydrophilic interaction chromatography. Anayticall Chem..

[85] Sugahara K., Mikami T., Uyama T., Mizuguchi S., Nomura K., Kitagawa H. (2003). Recent advances in the structural biology of chondroitin sulfate and dermatan sulfate. Curr. Opin. Struct. Biol..

[86] Kwok J., Warren P., Fawcett J. (2012). Chondroitin sulfate: A key molecule in the brain matrix. Int. J. Biochem. Cell Biol..

[87] Zou X., Foong W., Cao T., Bay B., Ouyang H., Yip G. (2004). Chondroitin sulfate in palatal wound healing. J. Dent. Res..

[88] Alliston T. (2010). Chondroitin sulfate and growth factor signaling in the skeleton: Possible links to mps vi. J. Pediatr. Rehabil. Med..

[89] Balazs E.A. (2009). Therapeutic use of hyaluronan. Struct. Chem..

[90] Gaffney J., Matou-Nasri S., Grau-Olivares M., Slevin M. (2010). Therapeutic applications of hyaluronan. Mol. BioSyst..

[91] Prestwich G.D. (2011). Hyaluronic acid-based clinical biomaterials derived for cell and molecule delivery in regenerative medicine. J. Control. Release.

[92] Khan F., Ahmad S.R. (2013). Polysaccharides and their derivatives for versatile tissue engineering application. Macromol. Biosci..

[93] Logesh A., Thillaimaharani K., Sharmila K., Kalaiselvam M., Raffi S. (2012). Production of chitosan from endolichenic fungi isolated from mangrove environment and its antagonistic activity. Asian Pac. J. Trop. Biomed..

[94] Khor E., Lim L.Y. (2003). Implantable applications of chitin and chitosan. Biomaterials.

[95] Rinaudo M. (2008). Main properties and current applications of some polysaccharides as biomaterials. Polym. Int..

[96] Chao Z., Ri-Fu Y., Tai-Qiu Q. (2013). Ultrasound-enhanced subcritical water extraction of polysaccharides from lycium barbarum l.. Sep. Purif. Technol..

[97] Song T., Pranovich A., Holmbom B. (2013). Separation of polymeric galactoglucomannans from hot-water extract of spruce wood. Bioresour. Technol..

[98] Cheng H., Feng S., Jia X., Li Q., Zhou Y., Ding C. (2013). Structural characterization and antioxidant activities of polysaccharides extracted from epimedium acuminatum. Carbohydr. Polym..

[99] Chen S., Chen H., Tian J., Wang J., Wang Y., Xing L. (2014). Enzymolysis-ultrasonic assisted extraction, chemical characteristics and bioactivities of polysaccharides from corn silk. Carbohydr. Polym..

[100] Abe M., Fukaya Y., Ohno H. (2010). Extraction of polysaccharides from bran with phosphonate or phosphinate-derived ionic liquids under short mixing time and low temperature. Green Chem..

[101] Do Nascimento D.M., Dias A.F., De Araújo Junior C.P., De Freitas Rosa M., Morais J.P.S., De Figueirêdo M.C.B. (2016). A comprehensive approach for obtaining cellulose nanocrystal from coconut fiber. Part II: Environmental assessment of technological pathways. Ind. Crops Prod..

[102] Matavire T.O. (March 2018). Extraction and Modification of Hemicellulose from Wheat Bran to Produce Entrapment Materials for the Controlled Release of Chemicals and Bioactive Substances. Master’s Thesis.

[103] Boukroufa M., Boutekedjiret C., Petigny L., Rakotomanomana N., Chemat F. (2015). Bio-refinery of orange peels waste: A new concept based on integrated green and solvent free extraction processes using ultrasound and microwave techniques to obtain essential oil, polyphenols and pectin. Ultrason. Sonochem..

[104] Maheshwari G., Sowrirajan S., Joseph B. (2017). Extraction and isolation of β-glucan from grain sources—A review. J. Food Sci..

[105] Amid B.T., Mirhosseini H. (2012). Optimisation of aqueous extraction of gum from durian (durio zibethinus) seed: A potential, low cost source of hydrocolloid. Food Chem..

[106] Yanuriati A., Marseno D.W., Harmayani E. (2017). Characteristics of glucomannan isolated from fresh tuber of porang (amorphophallus muelleri blume). Carbohydr. Polym..

[107] Zhang Y., Zhang Y., Taha A.A., Ying Y., Li X., Chen X., Ma C. (2018). Subcritical water extraction of bioactive components from ginseng roots (panax ginseng ca mey). Ind. Crops Prod..

[108] Guo Z., Lou Y., Kong M., Luo Q., Liu Z., Wu J. (2019). A systematic review of phytochemistry, pharmacology and pharmacokinetics on astragali radix: Implications for astragali radix as a personalized medicine. Int. J. Mol. Sci..

[109] Zhu W., Xue X., Zhang Z. (2016). Ultrasonic-assisted extraction, structure and antitumor activity of polysaccharide from polygonum multiflorum. Int. J. Biol. Macromol..

[110] Lv L., Cheng Y., Zheng T., Li X., Zhai R. (2014). Purification, antioxidant activity and antiglycation of polysaccharides from polygonum multiflorum thunb. Carbohydr. Polym..

[111] Alves A., Caridade S.G., Mano J.F., Sousa R.A., Reis R.L. (2010). Extraction and physico-chemical characterization of a versatile biodegradable polysaccharide obtained from green algae. Carbohydr. Res..

[112] Xu S.-Y., Huang X., Cheong K.-L. (2017). Recent advances in marine algae polysaccharides: Isolation, structure, and activities. Mar. Drugs.

[113] Yu X., Zhou C., Yang H., Huang X., Ma H., Qin X., Hu J. (2015). Effect of ultrasonic treatment on the degradation and inhibition cancer cell lines of polysaccharides from porphyra yezoensis. Carbohydr. Polym..

[114] Tsubaki S., Oono K., Hiraoka M., Onda A., Mitani T. (2016). Microwave-assisted hydrothermal extraction of sulfated polysaccharides from ulva spp. and monostroma latissimum. Food Chem..

[115] Lee W.-W., Ahn G., Wijesinghe W., Yang X., Ko C.-I., Kang M.-C., Lee B.-J., Jeon Y.-J. (2011). Enzyme-assisted extraction of ecklonia cava fermented with lactobacillus brevis and isolation of an anti-inflammatory polysaccharide. Algae.

[116] Rodriguez-Jasso R.M., Mussatto S.I., Pastrana L., Aguilar C.N., Teixeira J.A. (2011). Microwave-assisted extraction of sulfated polysaccharides (fucoidan) from brown seaweed. Carbohydr. Polym..

[117] Murray P.G., Grassick A., Laffey C.D., Cuffe M.M., Higgins T., Savage A.V., Planas A., Tuohy M.G. (2001). Isolation and characterization of a thermostable endo-β-glucanase active on 1, 3-1, 4-β-d-glucans from the aerobic fungus talaromyces emersonii cbs 814.70. Enzym. Microb. Technol..

[118] Nie S., Cui S.W., Xie M., Phillips A.O., Phillips G.O. (2013). Bioactive polysaccharides from cordyceps sinensis: Isolation, structure features and bioactivities. Bioact. Carbohydr. Diet. Fibre.

[119] Klein S. (2009). Polysaccharides in Oral Drug Delivery: Recent Applications and Future Perspectives.

[120] Olano-Martin E., Gibson G.R., Rastall R. (2002). Comparison of the in vitro bifidogenic properties of pectins and pectic-oligosaccharides. J. Appl. Microbiol..

[121] Witvrouw M., De Clercq E. (1997). Sulfated polysaccharides extracted from sea algae as potential antiviral drugs. Gen. Pharmacol. Vasc. Syst..

[122] Nangia-Makker P., Hogan V., Honjo Y., Baccarini S., Tait L., Bresalier R., Raz A. (2002). Inhibition of human cancer cell growth and metastasis in nude mice by oral intake of modified citrus pectin. J.Natl. Cancer Inst..

[123] Lattimer J.M., Haub M.D. (2010). Effects of dietary fiber and its components on metabolic health. Nutrients.

[124] Georgiev Y., Ognyanov M., Yanakieva I., Kussovski V., Kratchanova M. (2012). Isolation, characterization and modification of citrus pectins. J. BioSci. Biotechnol..

[125] Cheng H., Li S., Fan Y., Gao X., Hao M., Wang J., Zhang X., Tai G., Zhou Y. (2011). Comparative studies of the antiproliferative effects of ginseng polysaccharides on ht-29 human colon cancer cells. Med. Oncol..

[126] Jeon C., Kang S., Park S., Lim K., Hwang K.W., Min H. (2011). T cell stimulatory effects of korean red ginseng through modulation of myeloid-derived suppressor cells. J. Ginseng Res..

[127] Kim M.-H., Byon Y.-Y., Ko E.-J., Song J.-Y., Yun Y.-S., Shin T., Joo H.-G. (2009). Immunomodulatory activity of ginsan, a polysaccharide of panax ginseng, on dendritic cells. Korean J. Physiol. Pharmacol..

[128] Kim K.-H., Lee Y.-S., Jung I.-S., Park S.-Y., Chung H.-Y., Lee I.-R., Yun Y.-S. (1998). Acidic polysaccharide from panax ginseng, ginsan, induces th1 cell and macrophage cytokines and generates lak cells in synergy with ril-2. Planta Medica.

[129] Lee Y., Chung I., Lee I., Kim K., Hong W., Yun Y. (1997). Activation of multiple effector pathways of immune system by the antineoplastic immunostimulator acidic polysaccharide ginsan isolated from panax ginseng. Anticancer Res..

[130] Yoo D.-G., Kim M.-C., Park M.-K., Park K.-M., Quan F.-S., Song J.-M., Wee J.J., Wang B.-Z., Cho Y.-K., Compans R.W. (2012). Protective effect of ginseng polysaccharides on influenza viral infection. PLoS ONE.

[131] Sun Y. (2011). Structure and biological activities of the polysaccharides from the leaves, roots and fruits of panax ginseng ca meyer: An overview. Carbohydr. Polym..

[132] Lemmon H.R., Sham J., Chau L.A., Madrenas J. (2012). High molecular weight polysaccharides are key immunomodulators in north american ginseng extracts: Characterization of the ginseng genetic signature in primary human immune cells. J. Ethnopharmacol..

[133] Rigaud D., Ryttig K., Angel L., Apfelbaum M. (1990). Overweight treated with energy restriction and a dietary fibre supplement: A 6-month randomized, double-blind, placebo-controlled trial. Int. J. Obes..

[134] Birketvedt G., Aaseth J., Florholmen J., Ryttig K. (2000). Long-term effect of fibre supplement and reduced energy intake on body weight and blood lipids in overweight subjects. Acta Medica (Hradec Kralove).

[135] Pittler M.H., Ernst E. (2001). Guar gum for body weight reduction: Meta-analysis of randomized trials. Am. J. Med..

[136] Mueller-Cunningham W.M., Quintana R., Kasim-Karakas S.E. (2003). An ad libitum, very low-fat diet results in weight loss and changes in nutrient intakes in postmenopausal women. J. Am. Diet. Assoc..

[137] Hays N.P., Starling R.D., Liu X., Sullivan D.H., Trappe T.A., Fluckey J.D., Evans W.J. (2004). Effects of an ad libitum low-fat, high-carbohydrate diet on body weight, body composition, and fat distribution in older men and women: A randomized controlled trial. Arch. Int. Med..

[138] Howarth N.C., Saltzman E., Roberts S.B. (2001). Dietary fiber and weight regulation. Nutr. Rev..

[139] Birketvedt G.S., Shimshi M., Thom E., Florholmen J. (2005). Experiences with three different fi ber supplements in weight reduction. Med. Sci. Monit..

[140] Lyly M., Liukkonen K.-H., Salmenkallio-Marttila M., Karhunen L., Poutanen K., Lähteenmäki L. (2009). Fibre in beverages can enhance perceived satiety. Eur. J. Nutr..

[141] Lyly M., Ohls N., Lähteenmäki L., Salmenkallio-Marttila M., Liukkonen K.-H., Karhunen L., Poutanen K. (2010). The effect of fibre amount, energy level and viscosity of beverages containing oat fibre supplement on perceived satiety. Food Nutr. Res..

[142] Vitaglione P., Lumaga R.B., Montagnese C., Messia M.C., Marconi E., Scalfi L. (2010). Satiating effect of a barley beta-glucan–enriched snack. J. Am. Coll. Nutr..

[143] Vitaglione P., Lumaga R.B., Stanzione A., Scalfi L., Fogliano V. (2009). Β-glucan-enriched bread reduces energy intake and modifies plasma ghrelin and peptide yy concentrations in the short term. Appetite.

[144] Peters H.P., Boers H.M., Haddeman E., Melnikov S.M., Qvyjt F. (2008). No effect of added β-glucan or of fructooligosaccharide on appetite or energy intake. Am. J. Clin. Nutr..

[145] Gallaher D.D., Gallaher C.M., Mahrt G.J., Carr T.P., Hollingshead C.H., Hesslink  R., Wise J. (2002). A glucomannan and chitosan fiber supplement decreases plasma cholesterol and increases cholesterol excretion in overweight normocholesterolemic humans. J. Am. Col. Nutr..

[146] Chen H.-L., Sheu W.H.-H., Tai T.-S., Liaw Y.-P., Chen Y.-C. (2003). Konjac supplement alleviated hypercholesterolemia and hyperglycemia in type 2 diabetic subjects—A randomized double-blind trial. J. Am. Col. Nutr..

[147] Tappy L., Gügolz E., Würsch P. (1996). Effects of breakfast cereals containing various amounts of β-glucan fibers on plasma glucose and insulin responses in niddm subjects. Diabetes Care.

[148] Tapola N., Karvonen H., Niskanen L., Mikola M., Sarkkinen E. (2005). Glycemic responses of oat bran products in type 2 diabetic patients. Nutr. Metab. Cardiovasc. Dis..

[149] Mäkeläinen H., Anttila H., Sihvonen J., Hietanen R., Tahvonen R., Salminen E., Mikola M., Sontag-Strohm T. (2007). The effect of β-glucan on the glycemic and insulin index. Eur. J. Clin. Nutr..

[150] Maki K., Galant R., Samuel P., Tesser J., Witchger M., Ribaya-Mercado J., Blumberg J., Geohas J. (2007). Effects of consuming foods containing oat β-glucan on blood pressure, carbohydrate metabolism and biomarkers of oxidative stress in men and women with elevated blood pressure. Eur. J. Clin. Nutr..

[151] Hallfrisch J., Scholfield D.J., Behall K.M. (1995). Diets containing soluble oat extracts improve glucose and insulin responses of moderately hypercholesterolemic men and women. Am. J. Clin. Nutr..

[152] Nazare J.A., Normand S., Oste Triantafyllou A., Brac De La Perrière A., Desage M., Laville M. (2009). Modulation of the postprandial phase by β-glucan in overweight subjects: Effects on glucose and insulin kinetics. Mol. Nutr. Food Res..

[153] Battilana P., Ornstein K., Minehira K., Schwarz J., Acheson K., Schneiter P., Burri J., Jequier E., Tappy L. (2001). Mechanisms of action of β-glucan in postprandial glucose metabolism in healthy men. Eur. J. Clin. Nutr..

[154] Chen H.-L., Cheng H.-C., Liu Y.-J., Liu S.-Y., Wu W.-T. (2006). Konjac acts as a natural laxative by increasing stool bulk and improving colonic ecology in healthy adults. Nutrition.

[155] Wang L., Yu X., Yang X., Li Y., Yao Y., Lui E.M.K., Ren G. (2015). Structural and anti-inflammatory characterization of a novel neutral polysaccharide from north american ginseng (panax quinquefolius). Int. J. Biol. Macromol..

[156] Zhao H., Zhang W., Xiao C., Lu C., Xu S., He X., Li X., Chen S., Yang D., Chan A. (2011). Effect of ginseng polysaccharide on tnf-alpha and ifn-gamma produced by enteric mucosal lymphocytes in collagen induced arthritic rats. J. Med. Plant Res..

[157] Pillai R., Lacy P. (2011). Inhibition of neutrophil respiratory burst and degranulation responses by cvt-e002, the main active ingredient in cold-fx. Allergy Asthma Clin. Immunol..

[158] Biondo P.D., Goruk S., Ruth M.R., O’connell E., Field C.J. (2008). Effect of cvt-e002™(cold-fx^®^) versus a ginsenoside extract on systemic and gut-associated immune function. Int. Immunopharmacol..

[159] Wang M., Guilbert L.J., Li J., Wu Y., Pang P., Basu T.K., Shan J.J. (2004). A proprietary extract from north american ginseng (panax quinquefolium) enhances il-2 and ifn-γ productions in murine spleen cells induced by con-a. Int. Immunopharmacol..

[160] Wang M., Guilbert L.J., Ling L., Li J., Wu Y., Xu S., Pang P., Shan J.J. (2001). Immunomodulating activity of cvt-e002, a proprietary extract from north american ginseng (panax quinquefolium). J. Pharm. Pharmacol..

[161] Kim K.-S., Seo E.-K., Lee Y.-C., Lee T.-K., Cho Y.-W., Ezaki O., Kim C.-H. (2000). Effect of dietary platycodon grandiflorum on the improvement of insulin resistance in obese zucker rats. J. Nutr. Biochem..

[162] Han S.B., Park S.H., Lee K.H., Lee C.W., Lee S.H., Kim H.C., Kim Y.S., Lee H.S., Kim H.M. (2001). Polysaccharide isolated from the radix of platycodon grandiflorum selectively activates b cells and macrophages but not t cells. Int. Immunopharmacol..

[163] Li R., Qiu S., Chen H., Wang L. (2008). Immunomodulatory effects of astragalus polysaccharide in diabetic mice. J. Chin. Integr. Med..

[164] Chen W., Li Y.-M., Yu M.-H. (2008). Astragalus polysaccharides: An effective treatment for diabetes prevention in nod mice. Exp. Clin. Endocrinol. Diabetes.

[165] Chen W., Yu M., Li Y. Effects of astragalus polysaccharides on ultrastructure and oxidation/apoptosis related cytokines’ gene expression of non-obese diabetic mice’s islets. https://europepmc.org/abstract/cba/636484.

[166] Xue C., Yu G., Hirata T., Terao J., Lin H. (1998). Antioxidative activities of several marine polysaccharides evaluated in a phosphatidylcholine-liposomal suspension and organic solvents. Biosci. Biotechnol. Biochem..

[167] Oliveira J.T., Reis R. (2011). Polysaccharide-based materials for cartilage tissue engineering applications. J. Tissue Eng. Regen. Med..

[168] Wan A.C., Tai B.C. (2013). Chitin—a promising biomaterial for tissue engineering and stem cell technologies. Biotechnol. Adv..

[169] Croisier F., Jérôme C. (2013). Chitosan-based biomaterials for tissue engineering. Eur. Polymer J..

[170] Lu H.F., Lim S.-X., Leong M.F., Narayanan K., Toh R.P., Gao S., Wan A.C. (2012). Efficient neuronal differentiation and maturation of human pluripotent stem cells encapsulated in 3d microfibrous scaffolds. Biomaterials.

[171] Venkatesan J., Kim S.-K. (2010). Chitosan composites for bone tissue engineering—An overview. Mar. Drugs.

[172] Barud H.d.S., De Araújo Júnior A.M., Saska S., Mestieri L.B., Campos J.A.D.B., De Freitas R.M., Ferreira N.U., Nascimento A.P., Miguel F.G., Vaz M.M.d.O.L.L. (2013). Antimicrobial brazilian propolis (epp-af) containing biocellulose membranes as promising biomaterial for skin wound healing. Evid.-Based Complement. Altern. Med..

[173] Hrynyk M., Martins-Green M., Barron A.E., Neufeld R.J. (2012). Alginate-peg sponge architecture and role in the design of insulin release dressings. Biomacromolecules.

[174] Anilkumar T., Muhamed J., Jose A., Jyothi A., Mohanan P., Krishnan L.K. (2011). Advantages of hyaluronic acid as a component of fibrin sheet for care of acute wound. Biologicals.

[175] Liakos I., Rizzello L., Scurr D.J., Pompa P.P., Bayer I.S., Athanassiou A. (2014). All-natural composite wound dressing films of essential oils encapsulated in sodium alginate with antimicrobial properties. Int. J. Pharm..

[176] Gu Z., Xie H., Huang C., Li L., Yu X. (2013). Preparation of chitosan/silk fibroin blending membrane fixed with alginate dialdehyde for wound dressing. Int. J. Biol. Macromol..

[177] Singh R., Singh D. (2012). Radiation synthesis of pvp/alginate hydrogel containing nanosilver as wound dressing. J. Mate. Sci. Mater. Med..

[178] Anisha B., Biswas R., Chennazhi K., Jayakumar R. (2013). Chitosan–hyaluronic acid/nano silver composite sponges for drug resistant bacteria infected diabetic wounds. Int. J. Biol. Macromol..

[179] Mizrahy S., Peer D. (2012). Polysaccharides as building blocks for nanotherapeutics. Chem. Soc. Rev..

[180] Alvarez-Lorenzo C., Blanco-Fernandez B., Puga A.M., Concheiro A. (2013). Crosslinked ionic polysaccharides for stimuli-sensitive drug delivery. Adv. Drug Deliv. Rev..

[181] Reddy K., Krishna Mohan G., Satla S., Gaikwad S. (2011). Natural polysaccharides: Versatile excipients for controlled drug delivery systems. Asian J. Pharm. Sci..

[182] Jain A.K., Söderlind E., Viridén A., Schug B., Abrahamsson B., Knopke C., Tajarobi F., Blume H., Anschütz M., Welinder A. (2014). The influence of hydroxypropyl methylcellulose (hpmc) molecular weight, concentration and effect of food on in vivo erosion behavior of hpmc matrix tablets. J. Control. Release.

[183] Plackett D.V., Letchford K., Jackson J.K., Burt H.M. (2014). A review of nanocellulose as a novel vehicle for drug delivery. Nord. Pulp. Pap. Res. J..

[184] Kolakovic R., Peltonen L., Laukkanen A., Hirvonen J., Laaksonen T. (2012). Nanofibrillar cellulose films for controlled drug delivery. Eur. J. Pharm. Biopharm..

[185] Shelke N.B., James R., Laurencin C.T., Kumbar S.G. (2014). Polysaccharide biomaterials for drug delivery and regenerative engineering. Polym. Adv. Technol..

[186] Ji A., Su D., Che O., Li W., Sun L., Zhang Z., Yang B., Xu F. (2009). Functional gene silencing mediated by chitosan/sirna nanocomplexes. Nanotechnology.

[187] Chen M.-C., Huang S.-F., Lai K.-Y., Ling M.-H. (2013). Fully embeddable chitosan microneedles as a sustained release depot for intradermal vaccination. Biomaterials.

